# PITX2 gain-of-function mutation associated with atrial fibrillation alters mitochondrial activity in human iPSC atrial-like cardiomyocytes

**DOI:** 10.3389/fphys.2023.1250951

**Published:** 2023-11-13

**Authors:** Patrizia Benzoni, Lorenzo Da Dalt, Noemi Elia, Vera Popolizio, Alessandro Cospito, Federica Giannetti, Patrizia Dell’Era, Morten S. Olesen, Annalisa Bucchi, Mirko Baruscotti, Giuseppe Danilo Norata, Andrea Barbuti

**Affiliations:** ^1^ The Cell Physiology MiLab, Department Biosciences, Università degli Studi di Milano, Milano, Italy; ^2^ Department of Pharmacological and Biomolecular Sciences, Università degli Studi di Milano, Milano, Italy; ^3^ Cell Factory, Fondazione IRCCS Ca’Granda Ospedale Maggiore Policlinico, Milano, Italy; ^4^ Center for Cardiac Arrhythmias of Genetic Origin and Laboratory of Cardiovascular Genetics, Istituto Auxologico Italiano IRCCS, Milano, Italy; ^5^ Department of Molecular and Translational Medicine, Università degli Studi di Brescia, Brescia, Italy; ^6^ The Heart Centre, Rigshospitalet, Laboratory for Molecular Cardiology, Department of Cardiology, University Hospital of Copenhagen, Copenhagen, Denmark

**Keywords:** iPS-derived atrial cardiomyocytes, atrial fibrillation, PITX2, mitochondria, oxidative phosphorylation

## Abstract

Atrial fibrillation (AF) is the most common cardiac arrhythmia worldwide; however, the underlying causes of AF initiation are still poorly understood, particularly because currently available models do not allow in distinguishing the initial causes from maladaptive remodeling that induces and perpetuates AF. Lately, the genetic background has been proven to be important in the AF onset. iPSC-derived cardiomyocytes, being patient- and mutation-specific, may help solve this diatribe by showing the initial cell-autonomous changes underlying the development of the disease. Transcription factor paired-like homeodomain 2 (PITX2) has been identified as a key regulator of atrial development/differentiation, and the *PITX2* genomic locus has the highest association with paroxysmal AF. PITX2 influences mitochondrial activity, and alterations in either its expression or function have been widely associated with AF. In this work, we investigate the activity of mitochondria in iPSC-derived atrial cardiomyocytes (aCMs) obtained from a young patient (24 years old) with paroxysmal AF, carrying a gain-of-function mutation in *PITX2* (rs138163892) and from its isogenic control (CTRL) in which the heterozygous point mutation has been reverted to WT. PITX2 aCMs show a higher mitochondrial content, increased mitochondrial activity, and superoxide production under basal conditions when compared to CTRL aCMs. However, increasing mitochondrial workload by FCCP or β-adrenergic stimulation allows us to unmask mitochondrial defects in PITX2 aCMs, which are incapable of responding efficiently to the higher energy demand, determining ATP deficiency.

## 1 Introduction

Atrial fibrillation (AF) is the most common type of arrhythmia worldwide (Wijesurendra and Casadei, 2019; Kornej et al., 2020). AF is a complex disease; the mechanistic factors that determine its initial onset are multiple and, in most cases, unrecognizable (Nattel et al., 2020). Studies in patients and patient’s tissue samples provided insights into the pathophysiology of AF occurrence, but it is still difficult to distinguish the cell-autonomous abnormalities as primary disease drivers from the effects due to electrical and structural remodeling that cause AF self-maintenance (Nattel et al., 2020). Human-induced pluripotent stem cell-derived cardiomyocytes (hiPSC-CMs) represent a valuable and powerful model to study the molecular basis of AF since they carry the exact genetic background of the patient, are free from AF-dependent remodeling, and may thus recapitulate the early changes predisposing to arrhythmia.

Genomic analyses have long increased our understanding of the heritable nature of AF (Weng et al., 2017). The combination of large-scale genome-wide association studies (GWAS) and the study of rare cohorts with familial forms of AF have contributed to the identification of susceptibility genes and of common variants, some directly involving metabolism and mitochondrial function (Tao et al., 2016; Roselli et al., 2020). Variants located close to or within the paired-like homeodomain 2 (*PITX2*) genes on chromosome 4q25 have the highest association with AF (Bapat et al., 2018). Regardless of the presence of specific *PITX2* mutations, both under- and overexpression of *PITX2c*, the cardiac-specific isoforms have been found to be associated with AF in humans (Chinchilla et al., 2011; Pérez-Hernández et al., 2016). Noteworthy, genomic analyses have indicated that *PITX2* activates genes encoding the components of the electron transport chain and reactive oxygen species scavengers (Tao et al., 2016). It is well known that mitochondria play a central role in cardiomyocyte function as they produce energy to support the mechanical and electrical functions of the heart. ATP production is made possible by mitochondrial membrane potential through oxidative phosphorylation. This mechanism is also one of the main sources of reactive oxygen species (ROS) in the cell. Perturbations of these pathways and mitochondrial dysfunction have already been described in AF biopsies and in different models (Xie et al., 2015; Mason et al., 2020; Muszyński and Bonda, 2021; Pool et al., 2021). In this work, we describe, for the first time, *PITX2-*related changes in mitochondrial activity in human iPSC atrial cardiomyocytes (aCMs) derived from a young patient with paroxysmal AF carrying a gain-of-function mutation in *PITX2* (rs138163892) (Mora et al., 2017; Mechakra et al., 2019) when compared to its isogenic control.

## 2 Material and methods

### 2.1 iPS culture, differentiation, and atrial commitment validation

The hiPSC lines were derived from a 24-year-old patient diagnosed with paroxysmal AF carrying a heterozygous gain-of-function mutation in the *PITX2* gene (rs138163892). The point mutation was corrected by CRISPR-Cas9 obtaining the relative reverted clone. The hiPSC line with the gain-of-function mutation in *PITX2* is identified in the graphs as PITX2, while the reverted clone is identified as CTRL. The lines were cultured onto Matrigel-coated dishes in TeSR-E8 medium (STEMCELL Technologies). Cultures were maintained at 37°C at 5% CO_2_. Media were changed every other day. Cardiac differentiation was induced starting from the confluent monolayer of iPSC cultures using the STEMdiff Atrial Cardiomyocyte Differentiation Kit (STEMCELL Technologies). To validate the atrial commitment, the increases in specific atrial genes (*PITX2c*, *NR2F2*, and *KCN5A*) were tested (primer sequences are provided in Table 1). Cardiomyocytes obtained by a standard differentiation protocol (cardiomyocyte differentiation kit—Thermo Fisher Scientific) have been used as a reference, and three human auricles were used as a positive control.

**TABLE 1:** Primer sequences.

Gene	Primer forward	Primer reverse
***hGAPDH***	CAC​TCT​TCC​ACC​TTC​GAT​GC	GCC​GAG​TTG​GGA​TAG​GGC​CT
***hPITX2C***	CCG​CAG​AGA​AAG​ATA​AAA​GCC​A	GAT​TTC​TTC​GCG​TGT​GGA​CA
***hKCN5A***	TTC​TAC​CAC​CGG​GAA​ACG​GA	TTC​GGG​CAC​TGT​CTG​GAT​TC
***hNR2F2***	CCG​GGT​GGT​CGC​CTT​TAT​G	GCT​TTC​CAC​ATG​GGC​TAG​ATC

For functional analyses, hiPSC-derived aCMs or standard differentiated cardiomyocytes were isolated at day 21 of differentiation with trypsin-EDTA (Sigma-Aldrich) and plated on fibronectin-coated dishes (Corning). Around day 28, spontaneous contraction activity was recovered, and action potentials (APs) were recorded in the current clamp mode in the whole-cell configuration. Cells were superfused at physiological temperature (36°C ± 1°C) using Tyrode’s solution containing (mM) 137 NaCl, 5 KCl, 2 CaCl_2_, 1 MgCl_2_, 10 D-glucose, and 10 HEPES–NaOH; pH 7.4 with NaOH. Patch-clamp pipettes had a resistance of 5–7 MΩ when filled with the intracellular solution containing (mM) 120 KCl, 20 Na–HEPES, 10 MgATP, 0.1 EGTA–KOH, and 2 MgCl_2_; pH 7.1 with KOH. The following AP parameters were analyzed as follows: rate (beat per minutes-BPM), action potential amplitude (APA, mV), maximum diastolic potential (MDP, mV), and action potential duration at 30%–50% and 90% of repolarization (APD30, APD50, and APD90).

The atrial-specific ultra-rapid potassium current (I_Kur_) was measured in a high potassium extracellular solution (pH 7.4) containing (mM) 110 NaCl, 30 KCl, 1.8 CaCl_2_, 0.5 MgCl_2_, and 2.5 HEPES–NaOH. 4-Aminopyridine (4-AP; 50 μM) has been added to the extracellular solution to dissect 4-AP-sensitive I_Kur_ by subtracting the traces with 4AP from those without. Patch pipettes had a resistance of 4–7 MΩ when filled with the intracellular-like solution (pH 7.1) containing (mM) 120 KCl, 20 Na-HEPES, 10 MgATP, 0.1 EGTA–KOH, and 2 MgCl_2_. I_Kur_ was activated from a holding potential (hp) of −70 mV by applying 10-mV depolarizing voltage steps in the range −50/+50 mV long enough to reach steady-state activation. Steady-state current density was calculated as the ratio between the current intensity and cell capacitance at all voltages.

### 2.2 Seahorse analyses

CTRL and PITX2 aCMs at 21 days of differentiation were detached by enzymatic dissociation and seeded on a specific Seahorse XF cell culture plate (50.000 cells/well) previously coated using Matrigel. The assays were performed 7 days after seeding in order to restore the spontaneous beating activity of the aCM monolayer; this extra period of culture does not affect cell maturation. We evaluated oxygen consumption rates (OCRs) and extracellular acidification rates (ECARs) of live aCMs using the Seahorse XF24 extracellular flux analyzer (Agilent). The assay was performed in the recommended Seahorse XF DMEM medium at pH 7.4 and supplemented with 10 mM glucose, 2 mM L-glutamine, and 1 mM Na pyruvate.

During the assay, 1.5 µM oligomycin A (OligoA), an ATP synthase inhibitor; 0.5 µM rotenone/antimycin A (rot/AA), which inhibits complex I and III of the respiratory chain, respectively; and 0.5 µM FCCP that dissipates the mitochondrial proton gradient, are added to the media to evaluate the basal and maximal contribution of mitochondrial oxidative phosphorylation and glycolysis pathways. We mimicked potent sympathetic stimulation challenging aCMs using 1 µM isoprenaline during OCR and ECAR analyses. We quantify aCM basal and maximal respiration, and the spare capacity in both cell lines. ATP production rates from both mitochondrial respiration and glycolysis were estimated using a P/O ratio equal to 2.75 and a CO_2_ contribution factor of 0.6 for both lines, while the proton extrusion ratio (PER) is calculated directly using Seahorse Analytics software based on DMEM XF medium composition, multiplied by the microchamber volume (0,5 mL) and Kvol constant (1.1) (Romero et al., 2021).

The OCR and ECAR values were normalized against to the number of cells counted by Hoechst nuclei staining, followed by imaging recordings at the high-content screening platform (HCS, Molecular Instruments).

### 2.3 FACS analyses

CTRL and PITX2 aCMs at 21 days of differentiation were dissociated at single cells using TrypLE (Thermo Fisher Scientific) and diluted in PBS 0.5 mM EDTA and 10% FBS solution. aCMs were live-stained, following manufacturer’s guidelines using MitoTracker probes: MitoTracker Green (20 nM) to dissected total mitochondrial mass, MitoTracker Red CMXRos (100 nM) to dissect mitochondrial activity dependent upon hyperpolarized membrane potential, and MitoSOX Red (200 nM) to evaluate mitochondrial activity related to superoxide production and content in intermembrane space.

The intensity of fluorescence was investigated by flow cytometry analysis (Fortessa, Thermo Fisher Scientific), and data were presented as mean fluorescence intensity (MFI) normalized against control.

### 2.4 Molecular analysis of mitochondrial DNA and oxidative complexes

In this study, 28-day-old aCMs were solubilized in RIPA buffer adding protease inhibitors (Sigma-Aldrich) for protein analysis or in DNA extraction buffer (DNA extraction kit, QIAGEN) for mitochondrial DNA content quantification.

Western blot analysis was carried out loading 30 µg of whole-cell protein extracts separated by 12% SDS–PAGE and transferred onto PVDF membranes. Antibodies used were total OXPHOS Cocktail, anti-OPA-1, anti-βactin, anti-calnexin (1:1000; all from Abcam), and appropriate secondary antibodies, HRP (Jackson ImmunoResearch, 1:10000). For chemiluminescent acquisition, ChemiDoc system (Bio-Rad) was used after membrane incubation using the SuperSignal™ West Pico PLUS Chemiluminescent Substrate (Thermo Fisher Scientific).

The mitochondrial content was quantified by qPCR with the mtDNA/nDNA copy number assessment. The following primers were used to detect hmtDNA F: ACA​CCC​TCC​TAG​CCT​TAC​TA and R: GAT​ATA​GGG​TCG​AAG​CCG​C; hnDNA F: AGGGTATCTGGGCTCTGG and R: GGC​TGA​AAA​GCT​CCC​GAT​TAT.

### 2.5 Statistical analysis

The analyses were carried out in parallel between patient-derived aCMs and the isogenic control (n ≥ 3). Significant alterations have been delineated between the two groups by *p* < 0.05 with Student’s t-test comparison after checking for normal distribution of data with the Shapiro–Wilk test. More groups were analyzed with Levene’s test to check the homogeneity of variance and compared with one-way ANOVA, followed by pairwise comparison using Fisher’s test. *p* < 0.05 defines statistical significance.

When data were not normally distributed or with different variance, the Kolmogorov–Smirnov test was used to compare the data distribution. Significant differences in data distribution have been delineated by *p* < 0.05. Normally distributed data were reported as mean ± SEM. The median was reported when data were not normally distributed.

## 3 Results

In order to investigate a possible role of *PITX2* in mitochondrial activity in aCMs, we used human iPSC lines previously generated with a *PITX2* gain-of-function mutation (Mora et al., 2017) and the relative CRISPR-Cas9 reverted clone (CTRL) (see Supplementary Figure S1). We proved the atrial-like commitment of differentiated cardiomyocytes (aCMs) of both lines evaluating the expression of the following atrial markers: the orphan nuclear receptor COUP-TFII (*NR2F2* gene) known to confer atrial identity regulating the expression of many other atrial genes (Churko et al., 2018; Devalla et al., 2015; pin Wu et al., 2013); *PITX2c* gene encoding the cardiac isoform of paired-like homeodomain 2, involved in the development of specific cardiac left–right asymmetry, as well as in the regulation of atrial function (van Ouwerkerk et al., 2020); and the Kv1.5 channel encoded by the *KCN5A* gene, responsible for the atrial-specific ultra-rapid potassium current I_Kur_ (Wang et al., 1993).

**FIGURE 1:**
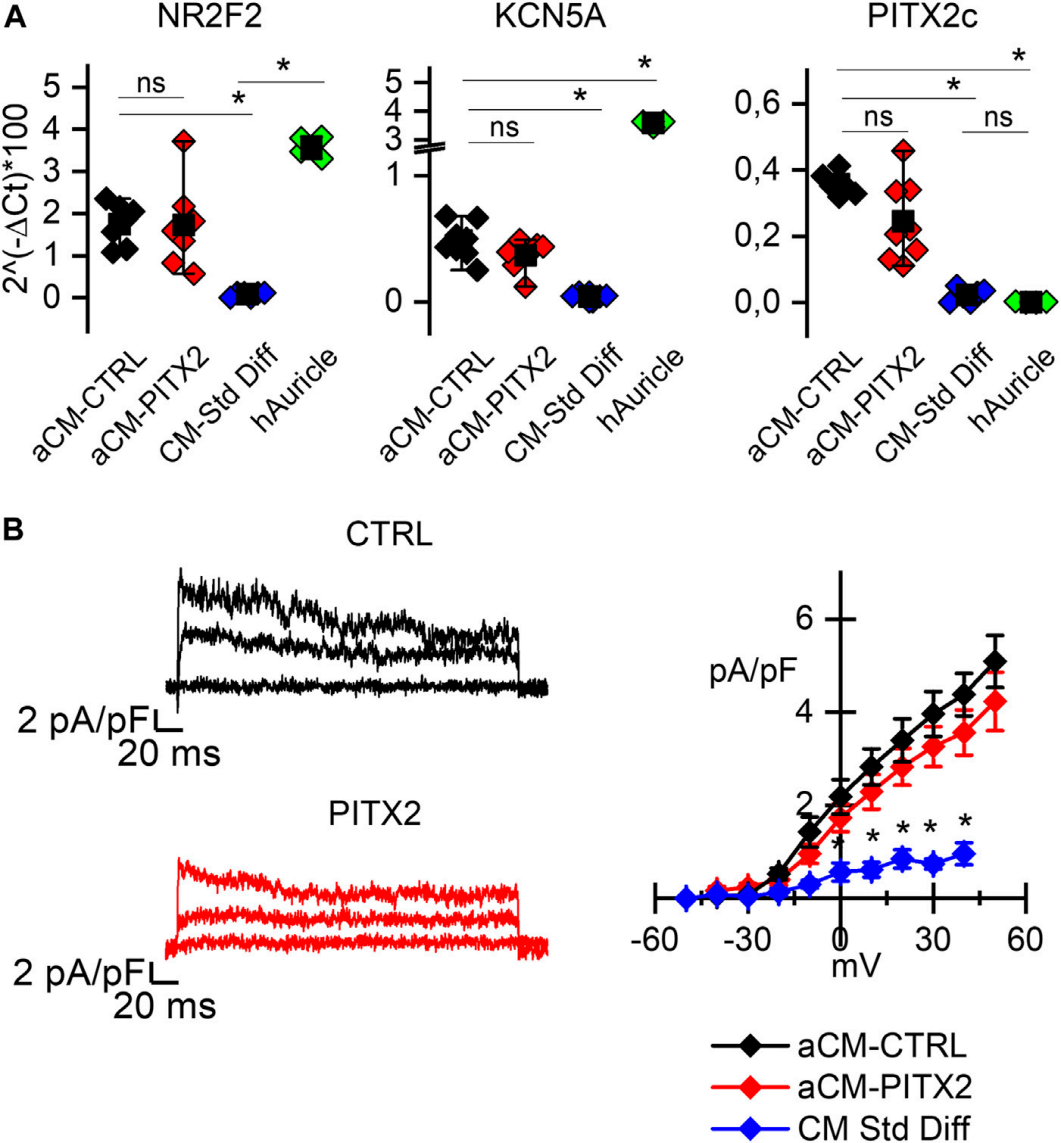
PITX2 mutation does not alter the atrial commitment of cardiomyocytes: **(A)** Box plots showing the expression of the atrial *NR2F2*, *KCNA5*, and *PITX2c* genes in atrial CMs derived from reverted CTRL (black dots; N = 6) and PITX2 line (red dots; N = 7), compared to the human atrial appendage (green dots; N = 4) and the CTRL cardiomyocytes obtained through a standard pan cardiac differentiation (blue dots; N = 4). Mean values ± SEM are reported as black squares and whiskers. **(B)** Representative 4-AP-sensitive traces and the plot of the mean I_Kur_ current density–voltage relation obtained from reverted CTRL (N = 14) and PITX2 aCMs (N = 13). CTRL cardiomyocytes obtained through a standard differentiation have been used as a negative control (N = 14) (colors as in A). Data are shown as mean ± SEM.

Figure 1A shows the comparison of the relative expression levels of all these genes in PITX2 and reverted CTRL aCMs against human atrial tissues, as a positive control (green dots), and cardiomyocytes obtained by a standard differentiation protocol, as a negative control (blue dots). Since the variance of the samples is different, the Kolmogorov–Smirnov test was used to compare the relative expressions among groups. The expression of *NR2F2* is significantly higher in aCMs than in the cardiomyocytes obtained from a standard differentiation, and it is similar when compared to the auricle samples; *PITX2c* expression is higher in aCMs than that in both standard differentiated cardiomyocytes and auricle tissues. To be noted, there is no difference between reverted CTRL and PITX2 aCMs in the *PITX2c* expression levels despite the presence of the mutation.

*KCN5A* instead is more highly expressed in auricles, but atrial differentiation causes its significant increase over standard differentiation. Indeed, from a functional point of view, the presence of the ultra-rapid potassium current (I_Kur_) can be recorded only after atrial-specific differentiation. In Figure 1B, representative 4-AP-sensitive I_Kur_ traces and the mean current-density plot are reported. PITX2 (red lines) and CTRL (black lines) aCMs show a higher ultra-rapid potassium current density than the cells differentiated under standard conditions (blue line/symbols). No difference between PITX2 and reverted CTRL is evident.

Previous data have shown that *PITX2c* overexpression is linked to oxidative stress, and this can contribute to the early developmental stage of AF (Tao et al., 2016; Collins et al., 2019; Mason et al., 2020); we focus our attention on mitochondrial dysfunction that could be linked to this specific gain-of-function mutation of *PITX2*.

**FIGURE 2:**
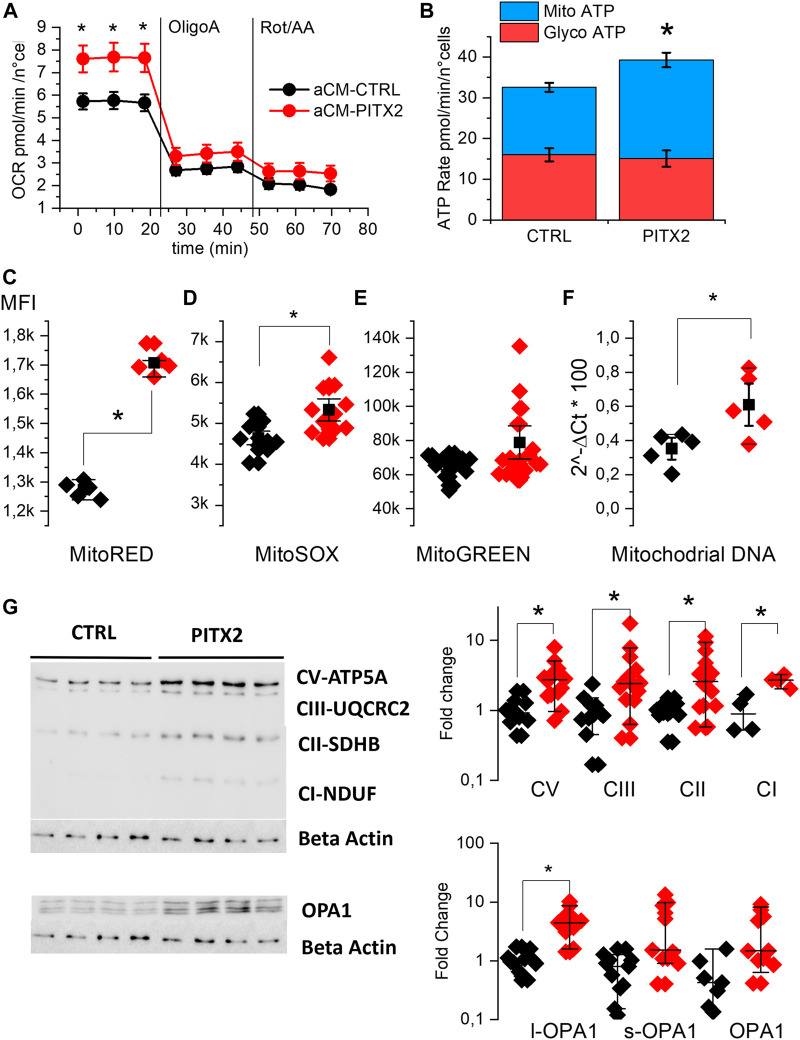
PITX2 mutation increases the basal mitochondrial activity of aCMs. **(A)** Mean oxygen consumption rate kinetic was measured by Seahorse XF real-time ATP rate assay in reverted CTRL (black, n = 20, N = 4) and PITX2 aCMs (n = 15, N = 4) at day 28 of differentiation. **(B)** Metabolic flux analysis showing quantification of mitochondrial ATP production (blue bars) and glycolytic ATP production (red bars) in reverted CTRL and PITX2 aCMs. Data are shown as mean ± SEM. **(C–E)** Box plot graph showing the mean fluorescence intensity recorded using MitoTracker RED **(C)** (CTRL N = 5 vs*.* PITX2 N = 5), MitoSOX **(D)** (CTRL N = 10 vs. PITX2 N = 11), and MitoTracker Green probes **(E)** (CTRL N = 15 vs*.* PITX2 N = 19) in CTRL (black dots) and PITX2 (red dots) samples. **(F)** Box plot graph showing mitochondrial DNA quantification by qPCR, color as shown in A (N = 5). Mean values ± SEM are reported as black squares and whiskers. **(G)** Western blot analyses and relative quantification of OXPHOS complexes and OPA-1 protein. β-Actin has been used as a reference protein (left panel; β-actin blot is re-used for illustrative purposes). Medians and 10–90 percentiles are reported as black lines and whiskers (right panels). Color as shown in **(A)**.

We evaluated OCR in aCMs at day 28 of differentiation using the Seahorse XF analyzer. Figure 2A shows the kinetic of this parameter. At basal condition, PITX2 aCMs show a significantly higher OCR (7.65 ± 0.35 pmol/min/n°cells) than CTRL aCMs (5.7 ± 0.21 pmol/min/n°cells), while after inhibition of complexes I and III, the difference disappeared. Consequently to the data collected, PITX2 aCMs show a different ATP production rate assuming an equal P/O ratio, as reported in Figure 2B. PITX2 aCMs show a higher mitochondrial ATP production (blue bars) (16.5 ± 1.1 vs. 24.2 ± 1.8 pmol/min/n°cells of Mito-ATP in CTRL and PITX2, respectively) compared to CTRL, while glycolytic ATP production (red bars) rates are equal in both cell lines (16.01 ± 1.6 vs. 15.1 ± 1.98 pmol/min/n°cells of Glyco-ATP in CTRL and PITX2, respectively).

To further evaluate the impact of the *PITX2* gain-of-function, the mitochondrial states in aCMs were investigated using MitoTracker probes and analyzed by flow cytometry immediately after the monolayer dissociation at day 21. Figures 2C–E report the mean fluorescent intensity recorded in different CTRL and PITX2 samples, dissecting the mitochondria with hyperpolarized membrane potential, mitochondrial superoxide production, and the total mitochondrial content. In line with the increase in mitochondrial ATP production, PITX2 aCMs present higher mitochondria with hyperpolarized membrane potential (Mito-RED CMXRos, 2C) and higher superoxide production (Mito-SOX, 2D).

Although it does not reach statistical significance, Mito-Green staining appears to be higher in PITX2, suggesting an increase in the mitochondrial content (Figure 2E). Indeed, the mitochondrial DNA content is significantly higher in PITX2 aCMs than in CTRL aCMs (Figure 2F).

Moreover, the Western blot analysis and relative protein quantifications of oxidative mitochondrial complexes (OXPHOS) reveal increased protein expression of all complexes in PITX2 aCMs than in CTRL cells, except for complex IV that is not detectable in both CTRL and PITX2 samples (Figure 2G and Supplementary Figure S4). In parallel, the long form of OPA-1 (OPA1-l), a protein involved in the fusion process of mitochondria, is upregulated (Figure 2G), suggesting more fused mitochondria and confirming the presence of more active and dynamic organelles. Coherently, the short form of OPA-1 (OPA1-s), known to promote mitochondrial fission, is not affected.

These data clearly suggest that the *PITX2* gain-of-function mutation increases the mitochondrial activity and content, oxygen consumption, and superoxide production under basal conditions.

This increase in mitochondrial activity cannot be accounted for by a different contraction activity between PITX2 and CTRL aCMs since these two groups have the same firing rate of spontaneous action potentials and there are no differences in the main AP parameters analyzed (Supplementary Figure S2).

**FIGURE 3:**
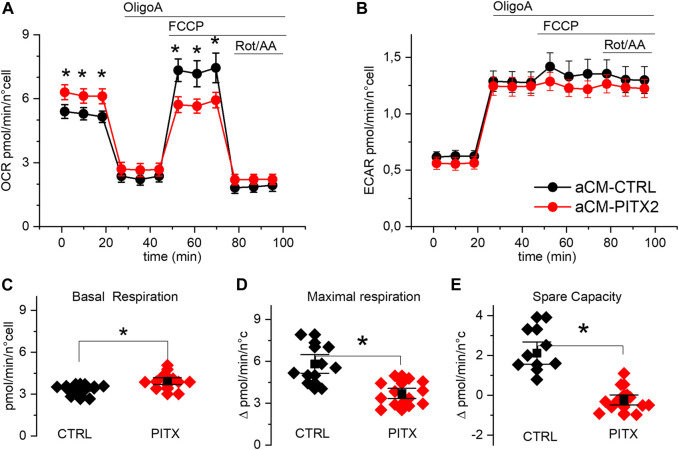
PITX2 aCMs show lower respiratory spare capacity: **(A, B)** mean oxygen consumption and acidification rate kinetics measured by Seahorse XF in reverted CTRL (n = 13, N = 3) and PITX2 aCMs (n = 12, N = 3) at day 28 of differentiation; FCCP was added to dissipate the proton gradient across the mitochondrial inner membrane and maximize respiration. **(C, D)** Box plot graphs showing the level of OCR values under basal conditions **(C)** and at the maximal level reached under FCCP **(D)**. **(E)** Box plot graph showing the difference between the maximal and basal respiration referred to as spare capacity. Mean values ± SEM are reported as black squares and whiskers.

**FIGURE 4:**
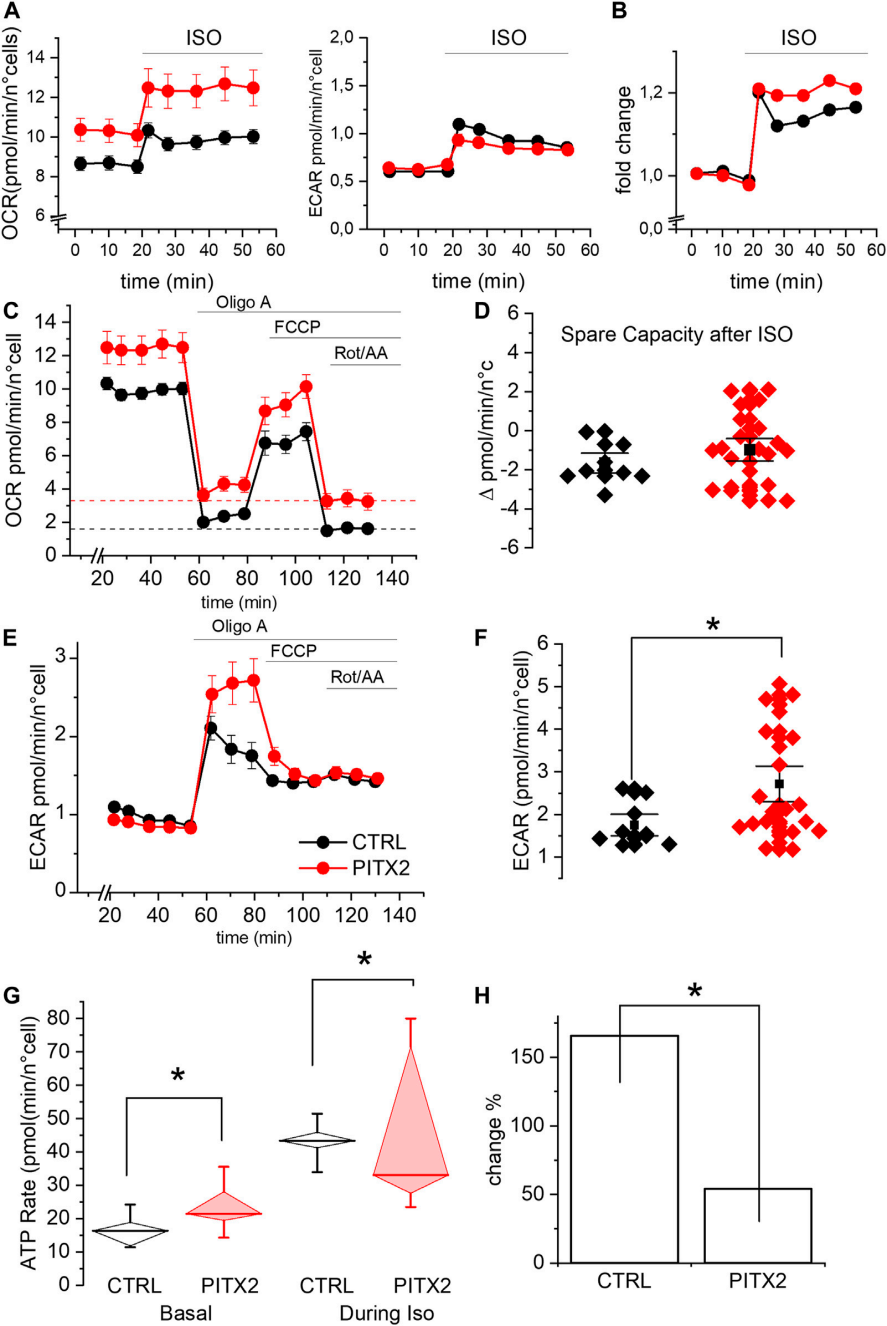
PITX2 mutation reduces the mitochondrial activity and ATP production under isoproterenol stimulation. **(A)** Mean oxygen consumption rate (left panel) and extracellular acidification rate (right panel) before and after isoproterenol (ISO) incubation in CTRL (black; n = 9, N = 3) and PITX2 aCMs (red; n = 23, N = 3) at day 28 of differentiation. **(B)** Fold change of the oxygen consumption rate induced by isoproterenol in CTRL and PITX2 aCMs (color as shown in A). **(C)** Mean oxygen consumption rate kinetic measured by Seahorse XF in CTRL (n9, N = 3) and PITX2 aCMs (n = 23, N = 3) at day 28 of differentiation under ISO (color as shown in A). Dash lines (color as shown in A) reported the mean values of non-mitochondrial oxygen consumption levels. **(D)** Box plot graph showing the spare capacity in CTRL aCMs (black) and PITX2 aCMs (red) after ISO treatment. Mean values ± SEM are reported as black squares and whiskers. **(E)** Extracellular acidification rate kinetic measured by Seahorse XF in CTRL (n = 9, N = 3) and PITX2 aCMs (n = 23, N = 3) at day 28 of differentiation under ISO. **(F)** Box plot graph showing the maximal ECAR levels reached by CTRL aCMs (black) and PITX2 aCMs (red) after ISO treatment. Mean values ± SEM are reported as black squares and whiskers. **(G)** Mitochondrial ATP production rate analysis at the basal condition and after ISO in CTRL (black) and PITX2 aCMs (red). Median values are shown as horizontal lines, and whiskers refer to 10–90 percentiles. **(H)** ISO-induced change in mitochondrial ATP production in CTRL and PITX2 aCMs.

To investigate the possible role of the *PITX2* mutation in the development of oxidative stress, we challenged basal mitochondrial activity with FCCP incubation (Figure 3) and with isoproterenol (ISO-1µM, Figure 4) known to increase aCM chronotropy and inotropy.

Mitochondrial uncoupling, with FCCP, strongly increased OCR (Figure 3A) in reverted CTRL aCMs but not in PITX2. Indeed, while basal respiration was higher in PITX2 aCMs (Figure 3C; CTRL: 3.81 ± 0.38 vs*.* PITX2: 4.83 ± 0.48 pmol/min/n cells), maximal uncoupled respiration was increased only in CTRL (Figure 3D; 5.81 ± 0.45 vs*.* 4.86 ± 0.69 pmol/min/n cells in CTRL and PITX2, respectively). The comparison of the spare capacity (maximal OCR under FCCP - basal OCR) between CTRL aCMs and PITX2 aCMs shows a higher respiration reserve in the former group (Figure 3E; 2.12 ± 0.38 in CTRL vs. −0.23 ± 0.17 in PITX2).

Unlike OCR, medium acidification (ECAR) increased similarly after OligoA injection and was not affected by mitochondrial uncoupling with FCCP in both cell lines (Figure 3B), meaning that the glycolytic pathway is not affected in PITX2 mutant cells. This finding strengthens the idea that in the presence of FCCP, the PITX2 mitochondria are unable to cope with the increased stressful condition, and this dysfunctional behavior may be a consequence of a higher vulnerability to oxidative damage produced by FCCP.

These results prompted us to investigate the effect of the *PITX2* gain-of-function on mitochondrial activity, following an adrenergic activation. Since an increased adrenergic stimulus is a well-known pro-arrhythmic condition (Benzoni et al., 2020; Piantoni et al., 2021) and the high frequency stimulation implies a high energy demand, we repeated the experiment by incubating the cells with the β-adrenergic agonist isoproterenol (ISO).

As shown in Figure 4A, both lines increase their OCR and ECAR once stimulated with ISO, as expected, given the increased energy demand. It should be noted that the OCR levels under ISO increased by a comparable fold change in both lines (Figure 4B). This result demonstrates that PITX2 cells retain the ability to respond to a physiological stimulus (ISO) but does not provide a logic framework to interpret the OCR differences, as shown in Figure 4A. A possible explanation for this phenomenon comes from the OCR kinetics described in Figure 4C, which clearly demonstrate that PITX2 aCMs have significantly higher non-mitochondrial oxygen consumption after rotenone/antimycin A injection (dotted lines, Figure 4C). Since OCR in the presence of rotenone/antimycin A is typically associated with the activity of enzymes that produce reactive oxygen species (Figure 4C), the OCR differences observed in Figures 4A,C are likely due to this non-mitochondrial oxygen consumption. Indeed, the differences in OCR between PITX2 and CTRL disappear when curves are normalized to their rotenone/antimycin A (Supplementary Figure S3). Taken together, these results suggest that PITX2 aCMs are more sensitive to oxidative stress induced by ISO.

Notably, under ISO incubation, mitochondrial uncoupling mediated by FCCP is not able to further increase oxygen consumption, not even in CTRL aCMs. This result suggests that the respiratory capacity of both PITX2 and CTRL aCMs can be positively recruited by ISO but cannot be further pumped by FCCP. Indeed, the spare capacity after ISO is negative in both cell lines (Figures 4C,D; median CTRL-2.03 vs*.* PITX2-1.02).

On the other hand, the ECAR kinetic graph reveals that β-adrenergic response activates the anaerobic pathway with lactic acid production, determining the acidification of the media in both CTRL and PITX2 aCMs (Figures 4A,E). Interestingly, after OligoA, PITX2 aCMs show higher increase in ECAR than CTRL cells, with a strong decrease in the pH level, demonstrating their need to activate more anaerobic pathways to be able to follow ISO stimulation and ATP demand (Figures 4E,F; median CTRL 1.54 vs. PITX2 2.13 pmol/min/n°cells).

To prove that the mitochondria are defective when challenged with an energy demand, we analyzed the rate of oxidative ATP production during ISO stimulation in CTRL and PITX2 aCMs comparing them with the one already reported under the basal condition (Figure 4G). During isoproterenol stimulation, mitochondrial ATP rates are significantly higher in CTRL aCMs than those in PITX2 aCMs, despite the high variability (median CTRL 43.33 vs. PITX2 33.03 pmol/min/n°cells). The fold change in oxidative ATP production from the basal condition to ISO is reported in Figure 4H to underline how the control is able to triplicate its ATP production through mitochondrial activity, while the PITX2 line cannot even double it.

## 4 Discussion

Most of the common genetic variants associated with AF are located within or close to the paired-like homeodomain 2 (*PITX2*) gene on chromosome 4q25 (Bapat et al., 2018; Roselli et al., 2020); and both under- and overexpression levels of *PITX2c* have been found in AF patients, regardless of specific mutations (Chinchilla et al., 2011; Pérez-Hernández et al., 2016; Syeda et al., 2017). However, a mechanistic explanation of how alterations in *PITX2* function increase vulnerability to atrial fibrillation in humans is still lacking.

The use of iPSC-derived cardiomyocytes is a recognized tool to model AF (Benzoni et al., 2020; Ramachandra et al., 2021; Ly et al., 2022; Schulz et al., 2023) and is beginning to be used to understand the physiology related to this transcription factor by generating knocked-out iPSC lines (Marczenke et al., 2017; Mun et al., 2022; Schulz et al., 2023). In this work, we used iPSC lines derived from a monogenic form of paroxysmal AF and its isogenic CTRL obtained by CRISP-Cas9 correction. iPSC showed a heterozygous mutation on *PITX2* (c.exon 6_A208G) (Mora et al., 2017) that causes the substitution of methionine for valine in the C-terminal, which corresponds to M207 in the cardiac-specific isoform *PITX2c* (Kirchhof et al., 2011). This same mutation was also described in the *PITX2c* isoform in a French family co-segregating with AF (Mechakra et al., 2019). The authors demonstrated that this specific mutation caused an increase in trans-activation activity of *PITX2c* in HeLa cells and HL-1 immortalized mouse atrial cell lines (Mechakra et al., 2019), and this evidence classifies the mutation as gain-of-function. Of note is the fact that the functional gain of the transcript is not due to an increase in its expression but to a larger potency in activating transcription.

To verify the contribution of *PITX2c* mutation on cardiomyocyte function in humans, it is critical to use a cellular model composed of aCMs. The use of a commercial kit dedicated to the differentiation toward this cardiac subtype allowed us to standardize and uniform the cardiomyocyte samples. As shown in Figure 1, aCMs express several atrial markers. Specifically, the cardiac isoform *PITX2c* is expressed equally by the mutated and the reverted CTRL lines, showing that the mutation *per se* did not alter the expression of the transcription factor.

Although these aCMs present I_Kur_, the atrial-specific current (Wang et al., 1993), at this maturation stage, cardiomyocytes still show a spontaneous contraction activity. The analysis of spontaneous action potentials (AP) revealed no difference in frequency and in AP parameters such as maximum diastolic potential, amplitude, and duration (Supplementary Figure S2). However, we cannot exclude the possibility that the increasing maturation state may unmask electrical defects. A recently published iPSC model with the NPPA-S64R mutation associated with AF also shows mitochondrial and electrical defects but only after cardiomyocyte maturation (Ly et al., 2022).

Since a transcriptional regulatory role of *PITX2c* on oxidative phosphorylation and the redox state of atrial CMs has been previously demonstrated in mice (Tao et al., 2016) and dysregulation of these pathways downstream to *PITX2c* seems to precede the onset of cardiac arrhythmia, at least in the zebrafish heart (Collins et al., 2019), we wondered if mitochondrial activity may be affected by the *PITX2*c gain-of-function mutation in human cardiomyocytes.

Given our interest specifically in mitochondrial activity, we analyzed oxidative phosphorylation complexes (OXPHOS) forming the electron transport chain and found them upregulated at the protein level in PITX2 aCMs (Figure 2G). In particular, complex I (referred to here as CI) or NADH:ubiquinone oxidoreductase is known to be directly regulated by *PITX2c* and is responsible for maintaining the inter-complex stability (Tao et al., 2016; Ly et al., 2022). Interestingly, we also found upregulated long-OPA-1 protein that controls the fusion of mitochondria, suggesting their different organization in PITX2 aCMs.

Using Seahorse XF, we analyzed the basal respiratory activity of CTRL and PITX2 aCMs and found that the latter presented an increased oxygen consumption rate; however, based on MitoTracker probes, PITX2 aCMs present increased mitochondria that are hyperpolarized and increased the superoxide content in intermembrane space. The increase in oxidative work is supported by increased mitochondrial content or organization, as suggested by mitochondrial DNA, and the increased expression of l-OPA-1 and OXPHOS complexes (Figures 2C–G). The increased OCR observed under the baseline condition (Figures 2, 3) may arise from mitochondrial oxidative overwork caused by the PITX2 mutation. An additional hypothesis is that the ATP demand in PITX2 cells is higher than normal; however, the reason for this increase is not yet clear since there is no evidence of increased contractility (AP shapes are identical in control and PITX2 cells). Whether other ATP consuming processes are also affected by the PITX2 mutation requires further investigation.

These data demonstrate for the first time the gain-of-function of the *M207V-PITX2c* mutation on OXPHOS in human atrial-like cardiomyocytes, in line with what was already described in mice and zebrafish hearts (Tao et al., 2016; Collins et al., 2019).

In our specific model, it remains to be defined how and why *PITX2* mutation induced an increase in oxidative respiration, an overwork of mitochondria. Since the values are basically higher in PITX2 aCMs, we wanted to investigate mitochondrial activity under conditions of increased energy demand and cell stress. We first applied FCCP, a substance that dissipates the proton gradient and consequently induces maximal cellular respiration. As shown in Figure 3, CTRL aCMs are able to activate their respiratory reserve increasing the oxygen consumption after FCCP incubation, while PITX2 aCMs remain at their basal level, with null spare capacity. This finding suggests that PITX2 mitochondria are at fault despite their baseline state. They are unable to adequately respond to energy stress due to an imbalance in energy supply and demand or because they are more vulnerable to the oxidative stress driven by FCCP. Cellular spare capacity can be influenced by a variety of circumstances (Marchetti et al., 2020). For instance, it has been shown that oxidative post-translational modification can control the enzymatic activity of OXPHOS, resulting in a reduction in cardiomyocyte spare capacity (Hill et al., 2010; Finley et al., 2011; Chen et al., 2022).

Then, we challenged aCMs with the β-adrenergic agonist isoproterenol (ISO) in order to increase the workload and consequently mitochondrial activity. Under this stimulus, both lines were able to increase OCR and ATP production (Figure 4), but PITX2 aCMs also show a strong increase in non-mitochondrial oxygen consumption dependent on ROS production. In this scenario, with an increased ROS production that affects the oxidative ATP production, the glycolytic pathway is activated more in order to compensate the ATP deficiency.

This experiment shows very clearly how even a gain in *PITX2c* functions is associated with a condition of impairment in counterbalancing energy stress.

Subsequent analyses will allow us to describe in a human context how this mitochondrial stress condition could be determined and how it could lead to arrhythmic susceptibility. One possible mechanism that could link mitochondrial defects and oxidative stress to arrhythmogenesis is defects in the coupling between cytosolic and mitochondrial Ca^2+^ increases (Kohlhaas et al., 2017; Mason et al., 2020; Lee et al., 2023).

Although the genetic forms of AF are rare and account for only a small fraction of all cases, the comprehension and description of underlying mechanisms predisposing to AF induction could be helpful in finding new druggable targets and provide a basis for understanding the multifactorial forms of AF.

## Data Availability

The original contributions presented in the study are included in the article/Supplementary Materials, further inquiries can be directed to the corresponding authors.
