# Supplementary material for: PITX2 gain-of-function mutation associated with atrial fibrillation alters mitochondrial activity in human iPSC atrial-like cardiomyocytes
Source: Front Physiol. 2023 Nov 13;14:1250951. doi: 10.3389/fphys.2023.1250951 (PMC10679737; doi:10.3389/fphys.2023.1250951)
Supplement: Supplementary file 1 [file Table1.DOCX]

Supplementary Material

PITX2 gain of function mutation associated with Atrial Fibrillation alter mitochondrial activity in human iPSC atrial-like cardiomyocytes.

Patrizia Benzoni^*^, Lorenzo Da Dalt, Noemi Elia, Vera Popolizio, Alessandro Cospito, Federica Giannetti, Patrizia Dell’Era, Morten S. Olesen, Annalisa Bucchi, Mirko Baruscotti, Danilo Norata, Andrea Barbuti^1^.

*** Correspondence:** Corresponding Authors

Patrizia Benzoni email:patrizia.benzoni@unimi.it

Andrea Barbuti email:andrea.barbuti@unimi.it

# Supplementary Figure 1

Supplementary Figure 1.

**PITX2-reverted CTRL**

**PITX2-Patient line**


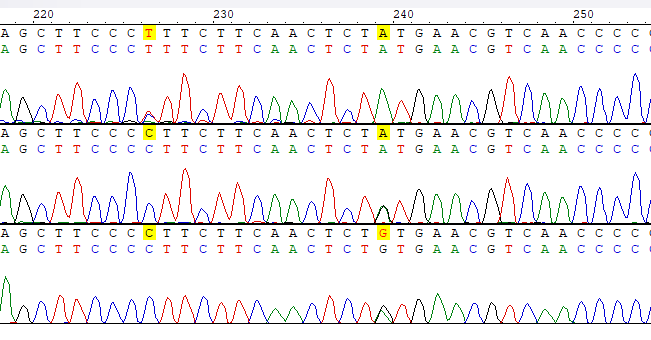

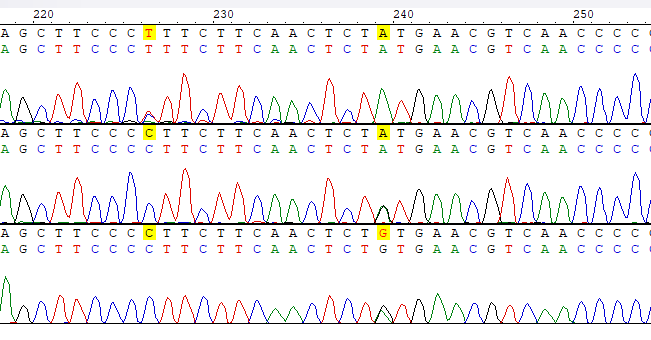


**Supplementary Figure 1*. PITX2 gene sequencing in hiPS-PITX2 and reverted CTRL lines***. A) Sanger sequencing of the regions in the DNA of patient-derived iPSC containing the heterozygous mutations in PITX2, and in the reverted control iPSC cells in which the mutation was corrected


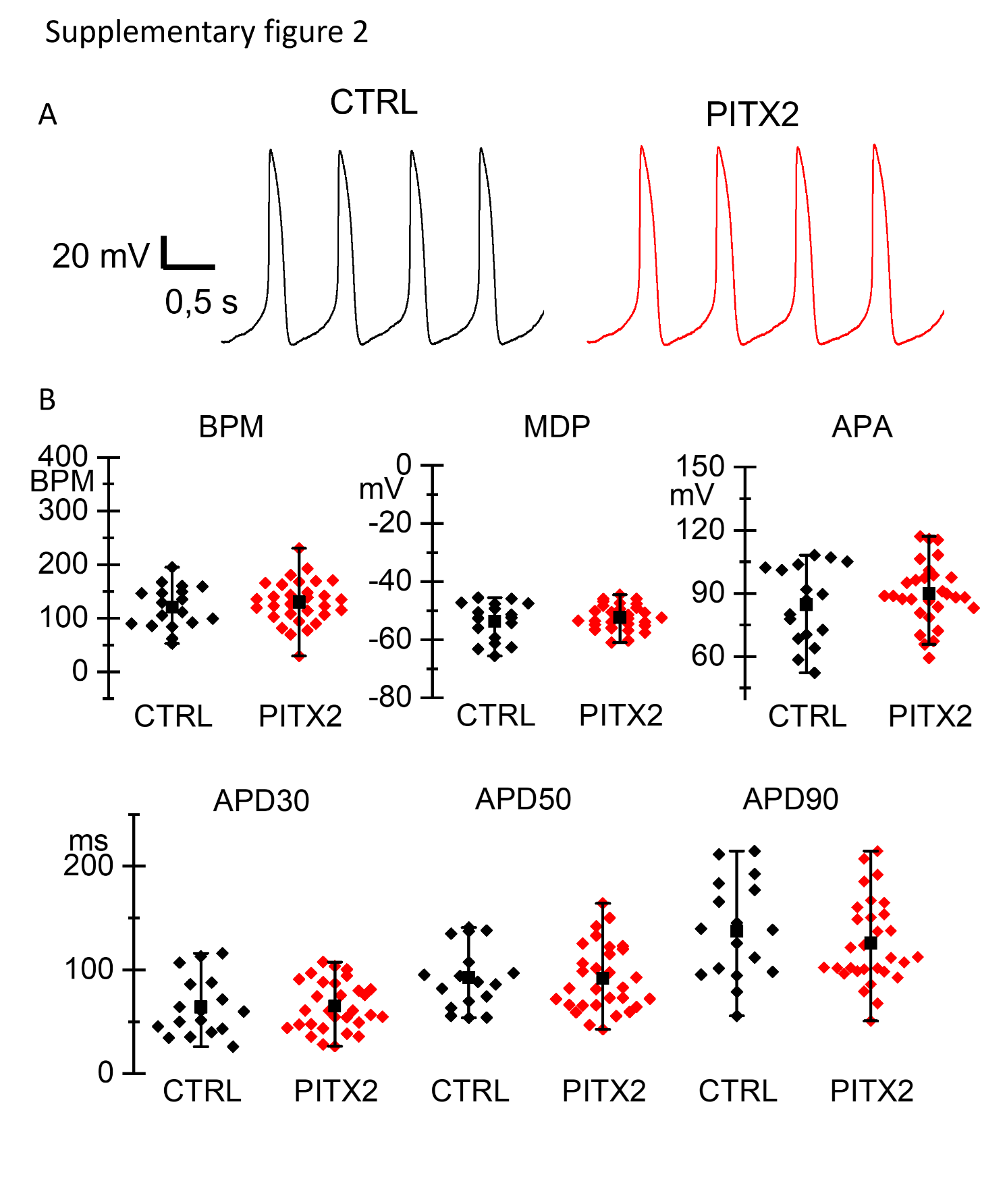


**Supplementary Figure 2. *PITX2 mutation does not alter spontaneous action potential parameters.***

A) Representative voltage traces of spontaneous action potential recorded from CTRL and PITX2 aCM at day 28 of differentiation, as indicated. B) Scatter plots of the firing rate (beats per minutes, BPM), maximum diastolic potential (MDP), action potential amplitude (APA), and action potential duration at 30%, 50% and 90% of repolarization (APD30, APD50 and APD90, respectively) in CTRL (black diamonds; N=17) and PITX2 (red diamonds; N=29). Mean ± SEM values are reported as black squares and whiskers.


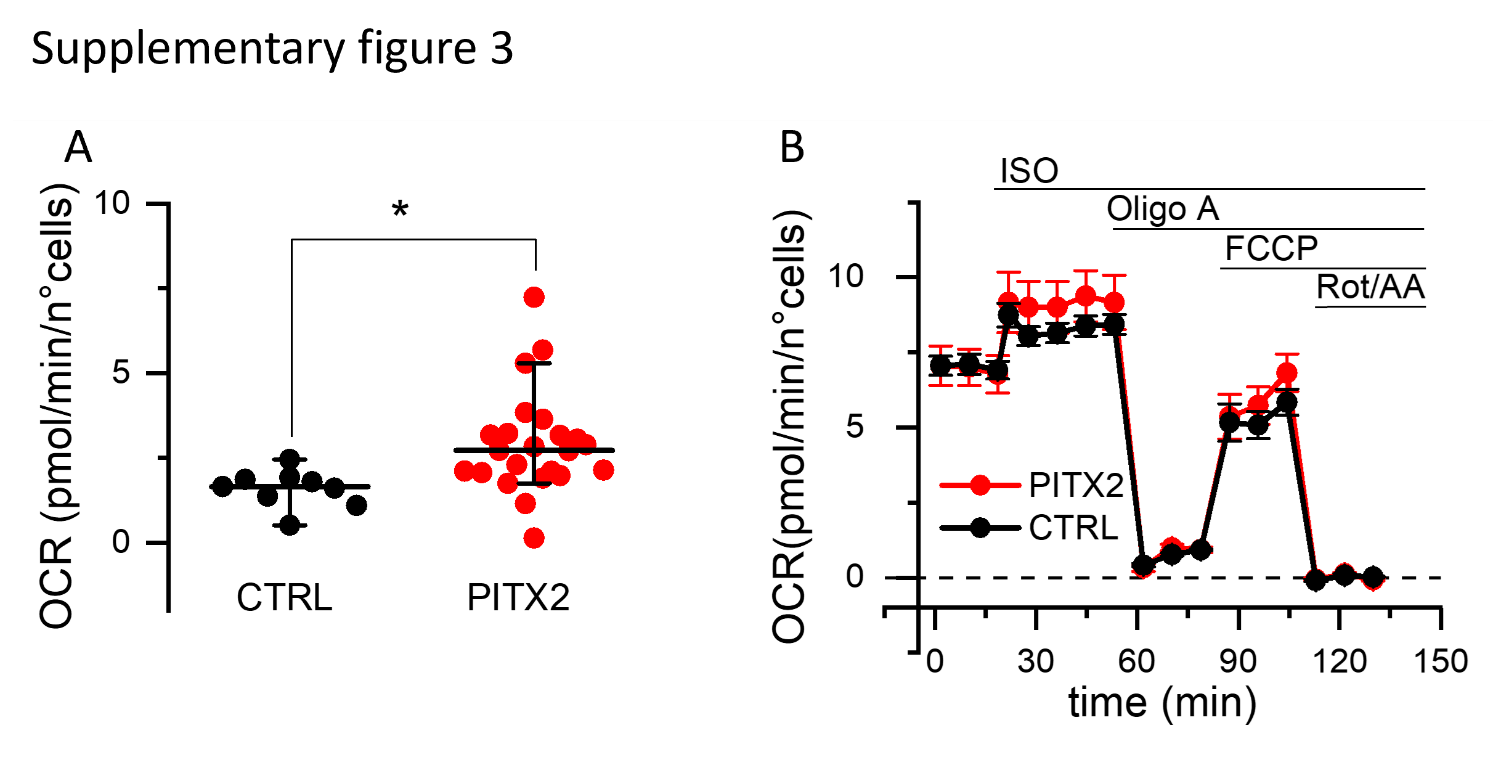


**Supplementary figure 3. *PITX2 mutation increases non-mitochondrial oxygen consumption under Isoproterenol stimulation.*** A) Box plot graphs showing the level of non-mitochondrial OCR values under Isoproterenol after the injection of Rotenone/Antimycin A. Medians and 10-90 percentile are reported as black lines and whiskers. B) Mean oxygen consumption rate kinetics measured by Seahorse XF in CTRL (Black line; n=9, N=3) and PITX2 aCM (Red line; n=23, N=3) at day 28 of differentiation under ISO in which non-mitochondrial oxygen consumption levels were subtracted and imposed at 0 (dash line).


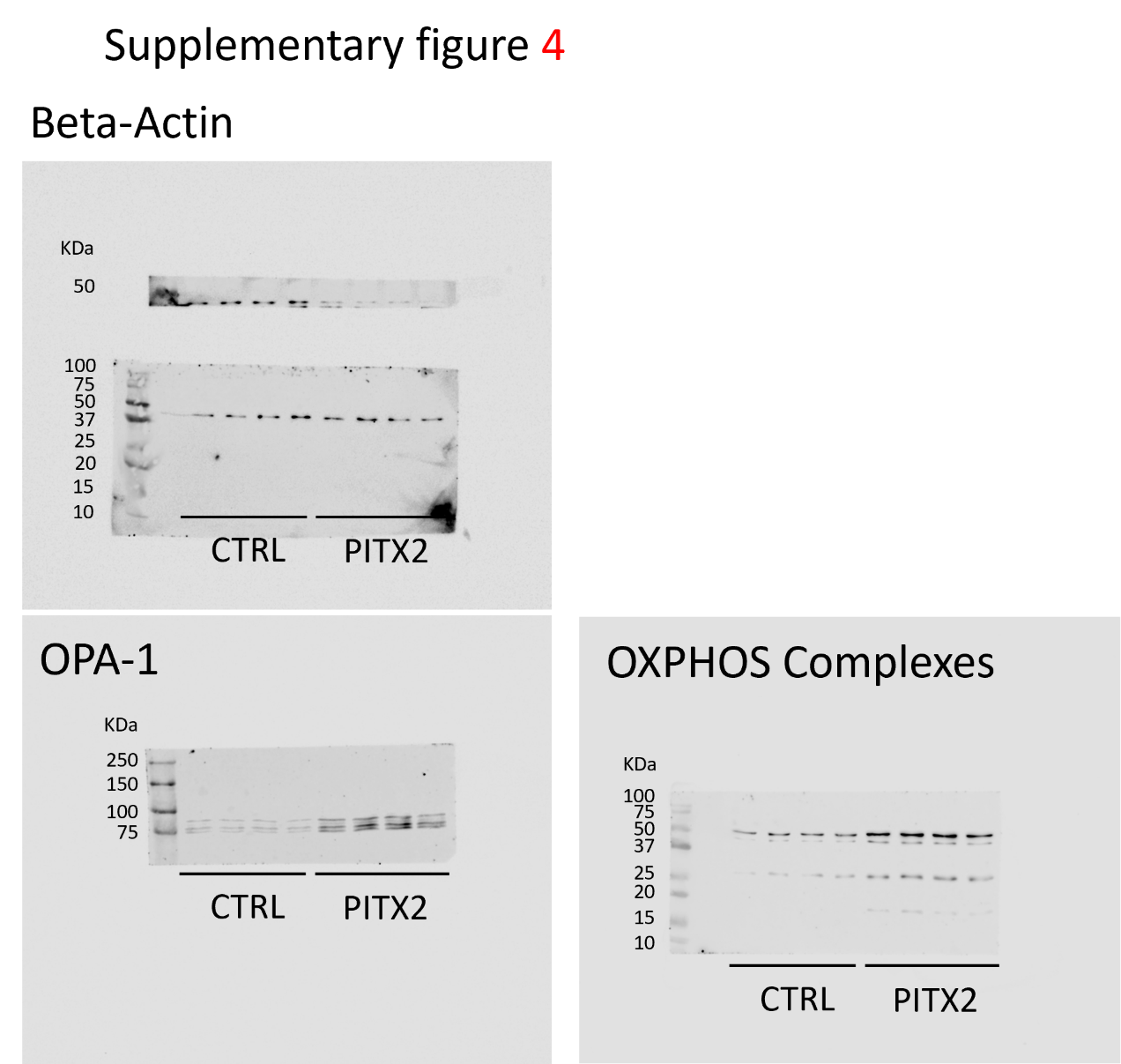


**Supplementary figure 4. *Representative Western blot for OXPHOS complexes and OPA-1.*** A full scan of the entire original blots presented in figure 2G. For β-actin two different acquisitions of the same samples are shown, the second being the one selected and shown in Figure 2G. Samples are re-loaded with the same amount of total protein and run at the same time tha.n OXPHOS complexes.
